# Ferroptosis: roles and molecular mechanisms in diabetic cardiomyopathy

**DOI:** 10.3389/fendo.2023.1140644

**Published:** 2023-04-20

**Authors:** Yangting Zhao, Binjing Pan, Xiaoyu Lv, Chongyang Chen, Kai Li, Yawen Wang, Jingfang Liu

**Affiliations:** ^1^ The First Clinical Medical College, Lanzhou University, Lanzhou, Gansu, China; ^2^ Department of Endocrinology, The First Hospital of Lanzhou University, Lanzhou, Gansu, China

**Keywords:** diabetes mellitus, diabetic cardiomyopathy, ferroptosis, iron metabolism, oxidative stress

## Abstract

Diabetic cardiomyopathy (DCM) is a serious complication of type 1 and type 2 diabetes, which leads to the aggravation of myocardial fibrosis, disorders involving systolic and diastolic functions, and increased mortality of patients with diabetes through mechanisms such as glycolipid toxicity, inflammatory response, and oxidative stress. Ferroptosis is a form of iron-dependent regulatory cell death that is attributed to the accumulation of lipid peroxides and an imbalance in redox regulation. Increased production of lipid reactive oxygen species (ROS) during ferroptosis promotes oxidative stress and damages myocardial cells, leading to myocardial systolic and diastolic dysfunction. Overproduction of ROS is an important bridge between ferroptosis and DCM, and ferroptosis inhibitors may provide new targets for the treatment of patients with DCM.

## 1 Introduction

Diabetes mellitus is a chronic metabolic disorder caused by a combination of genetic and environmental factors, and its prevalence is increasing annually. The global prevalence of diabetes in people aged 20-79 years is currently estimated to be 10.5% (536.6 million people) and is expected to rise to 12.2% (783.2 million people) by 2045. There are currently 141 million adults with diabetes in China, and it is expected that by 2045, more than 174 million people will have diabetes (1).

Patients with diabetes are at a significantly higher risk of cardiovascular comorbidities owing to long-term metabolic disorders, persistent high glucose status, and insulin resistance (IR). Diabetic cardiomyopathy (DCM), a specific form of heart disease, has garnered considerable attention. DCM, also known as non-vascular myocardial dysfunction, refers to the development of abnormal myocardial structures and clinical manifestations in patients with diabetes in the absence of other cardiac risk factors, such as coronary artery disease, hypertension, and severe valvular disease (2).

There is still a lack of clear expert consensus on the definition of DCM, and its prevalence and incidence remain unclear. It has been estimated that early features of DCM can be observed in one-quarter to one-third of asymptomatic patients with diabetes. Among them, 15.5% with type 1 diabetes mellitus (T1DM) have abnormal myocardial systolic or diastolic function, whereas patients with type 2 diabetes mellitus (T2DM) are mostly middle-aged and elderly, with a relatively higher risk of cardiovascular system complications (3). A prospective study showed that the prevalence of DCM in subjects diagnosed with clinical or preclinical stages of DCM was approximately 38%, with a prevalence of 48% in female patients, mostly older than 65 years (4). DCM is also a major cause of heart failure (HF) in diabetic patients, and the risk of HF in patients with diabetes increases by 74% (5). The incidence of myocardial ischemia is 2.45 to 2.99 times that of non-diabetic patients (6). DCM is the leading cause of increased mortality among patients with diabetes in both developed and developing countries, and its prevalence increases with the incidence of obesity and T2DM, thereby placing a serious burden on the global economy and health management systems.

Ferroptosis is a new type of programmed cell death that is often accompanied by massive iron accumulation and lipid peroxidation, as well as a deficiency in oxidoreductases, especially glutathione peroxidase 4 (GPX_4_) (6, 7). In 2012, Dixon et al. named this death pattern of iron-dependent non-apoptotic cell death as “ferroptosis” (8). Ferroptosis can affect GPX_4_ directly or indirectly through different pathways, causing a decrease in cellular antioxidant capacity and accumulation of lipid reactive oxygen species (ROS), ultimately leading to cell death (7).

In recent years, studies have shown that ferroptosis is strongly associated with various diseases including ischemic heart disease, kidney disease, liver damage, degenerative diseases, and diabetes (6, 9). Ferroptosis inhibitors can improve the progression of DCM-related pathologies and may be used as novel therapeutic modalities for DCM treatment (10). This paper reviews the relationship and molecular mechanisms of ferroptosis and DCM to provide new ideas and targets for the treatment of DCM.

## 2 Roles and regulatory mechanisms of ferroptosis

### 2.1 Ferroptosis and iron metabolism

Iron is an important nutrient in the human body and is involved in a variety of biological processes, such as oxygen transport, lipid metabolism, DNA and protein synthesis, and cellular respiration. Abnormalities in the content or distribution of iron in the body may lead to the occurrence and progression of certain diseases (11). Intracellular iron metabolism mainly involves iron absorption, export, utilization, and storage, and an imbalance between these processes may affect the susceptibility of cells to ferroptosis (12). On the one hand, the redox properties of iron make free Fe^2+^ prone to undergo Fenton reactions with lipid peroxides, which produces a highly toxic hydroxyl radical and induces a strong oxidative stress response, which is a core mechanism for ferroptosis. On the other hand, Fe^2+^ and Fe^3+^ are also cofactors that can enhance the activities of various metabolic enzymes, resulting in the formation of free radicals such as alkoxy (RO) and peroxy (RO_2_), promoting lipid peroxidation, and inducing ferroptosis (13, 14). In addition, the degradation of ferritin and subsequent autophagy can increase the intracellular unstable iron content and enhance sensitivity to ferroptosis. Iron regulatory protein 1 (IRP_1_) and iron regulatory protein 2 (IRP_2_) regulate intracellular iron storage, release, entry, and exit, thereby maintaining cellular iron homeostasis. Changes in their levels may affect the amount of unstable iron in cells, thereby altering the sensitivity of cells to ferroptosis (15, 16). Therefore, an abnormal iron metabolism is necessary for ferroptosis.

### 2.2 Characteristics of ferroptosis

Ferroptosis is mainly characterized by iron overload, which causes substantial lipid peroxide production and, ultimately, cell death (9). Ferroptosis is morphologically, biochemically, and genetically different from cell death by apoptosis, necrosis, and autophagy. Ferroptosis is morphologically characterized by the contraction of mitochondria, reduction in volume, increased membrane density, and the reduction or disappearance of mitochondrial cristae. However, the cell nuclei are normal in size, and there is no chromatin condensation. This is an essential distinction from other cell death modalities such as apoptosis and necrosis. Ferroptosis is biochemically predominantly iron-dependent and is mainly characterized by an increase in Fe^2+^ concentration and lipids. Excessive production of ROS by Fe^2+^ through Fenton reactions causes intracellular lipid peroxidation, whereas iron enhances lipoxygenase (LOX) activity to promote ferroptosis (17). Genetically, ferroptosis is a biological process regulated by multiple genes, and a variety of genes involved in iron metabolism, lipid synthesis, and oxidative stress regulation can modulate ferroptosis (7).

### 2.3 Mechanism of ferroptosis

In addition to iron-dependent cell death, ferroptosis is caused by an imbalance between the production and degradation of intracellular lipid ROS and is regulated by multiple metabolic and redox systems. ROS are mainly produced by the iron-dependent Fenton reaction and mitochondrial nicotinamide adenine dinucleotide phosphate (NADPH) oxidases (NOXs) family enzymes. Mitochondria are the metabolic centers of most mammalian cells, and are important sources of ROS. Excessive ROS may result in oxidative damage to mitochondrial proteins and DNA, which impairs mitochondrial function, eventually leading to ferroptosis (18).

The system Xc^-^ -glutathione (GSH)-GPX_4_ axis plays a central role in limiting lipid peroxidation. Ferroptosis is associated with the inactivation of cellular antioxidant systems, especially system Xc^-^, which causes the accumulation of lipid peroxides (17). System Xc^-^ is an amino acid counter-transport protein widely distributed in phospholipid bilayers and consists of two independent proteins: solute carrier family 7 member 11 (SLC_7_A_11_) and solute carrier family 3 member 2 (SLC_3_A_2_) (19). GPX is the main endogenous mechanism that prevents peroxidation, among which GPX_4_ is a key regulator of ferroptosis and inhibits ferroptosis by inhibiting the production of lipid peroxides. GSH is an essential cofactor for GPX_4_, which converts reduced GSH to oxidized GSH (GSSG) and reduces toxic lipid peroxides (L-OOH) to non-toxic alcohols (L-OH), thereby decreasing oxidative stress damage and inhibiting the onset of ferroptosis (7, 20).

Cystine and glutamate are exchanged in and out of the cell *via* system Xc^-^ at a ratio of 1:1. System Xc^-^ transports extracellular cystine into the cell and converts it to cysteine, which is then used for GSH synthesis. GSH reduces ROS and reactive nitrogen species through the action of GPX (21). Thus, the inhibition of system Xc^-^ activity can affect GSH synthesis by inhibiting cystine uptake, contributing to a reduction in GPX activity, a decrease in cellular antioxidant capacity, accumulation of lipid ROS, and ultimately the occurrence of oxidative damage and ferroptosis.

Ubiquinone, also known as coenzyme Q_10_ (CoQ_10_), is an electron carrier in the mitochondrial respiratory chain and a potent lipophilic antioxidant (22). Ubiquinol (CoQ_10_H_2_) is the reduced form of coenzyme Q_10_ that reduces oxidative stress, inhibits adipocyte differentiation, and suppresses lipid ROS accumulation (20). Mitochondrial CoQ_10_ inhibits apoptosis, whereas non-mitochondrial CoQ_10_ prevents ferroptosis. Ferroptosis suppressor protein 1 (FSP_1_), a flavoprotein oxidoreductase, may determine the role of CoQ_10_ in apoptosis and ferroptosis, and its pro-apoptotic function may be achieved through the inhibition of redox activity (18). FSP_1_, originally named apoptosis-inducing factor mitochondrial associated 2 (AIFM_2_), is also a mitochondrial pro-apoptotic protein (23). It has been found that FSP_1_ is able to use NADPH to regenerate lipophilic radicals to trap antioxidant; that is, FSP_1_ is responsible for regenerating CoQ_10_. FSP_1_ shuttles the reducing equivalents from NADPH to the lipid bilayer, allowing CoQ_10_ to interconvert with CoQ_10_H_2_, thereby inhibiting lipid peroxidation and maintaining the effective concentration of CoQ_10_H_2_. The FSP_1_-CoQ_10_-NADPH pathway is an independent parallel system, also known as the non-dependent GSH/GPX_4_ ferroptosis inhibition pathway, which is the main pathway of endogenous antioxidant enzyme regeneration, and synergistically inhibits lipid peroxidation and ferroptosis with GPX_4_ and GSH (24). Tetrahydrobiopterin (BH_4_) is an endogenous antioxidant that protects cells from ferroptosis by reducing lipid peroxidation. Dihydrobiopterin (BH_2_) can be reduced to BH_4_ by dihydrofolate reductase (DHFR) (25); therefore, DHFR is also an important regulator of ferroptosis.

Lipid peroxidation is a prerequisite for ferroptosis and is closely associated with lipid metabolism. Fatty acids (FA) are important components of cellular lipid metabolism and have a variety of cellular functions, including energy supply, cell membrane formation, and function as precursors to a variety of signaling molecules (26). The accumulation of polyunsaturated fatty acids (PUFA), such as arachidonic acid (AA), and the reduction in lipid peroxide scavenging capacity lead to ferroptosis (6). It has been shown that phosphatidyl ethanolamine (PE) containing AA or its derivative, adrenic acid (ADA), is a key phospholipid in the induction of cellular ferroptosis. Acyl CoA synthase long-chain family member 4 (ACSL_4_) and lysophosphatidylcholine acyltransferase 3 (LPCAT_3_) are two key enzymes involved in the synthesis of PE, activating PUFA and affecting their transmembrane properties. PUFA can be acylated by ACSL_4_ to form polyunsaturated fatty acid acyl-coenzyme A (PUFA-CoA), which is then esterified by LPCAT_3_ and finally reacts with PE to form PUFA- phosphatidyl ethanolamines (PUFA-PEs). PUFA-PEs can be oxidized by LOX to L-OOH products such as PE-AA-OOH or PE-ADA-OOH. Therefore, reducing the expression of ACSL_4_ and LPCAT_3_ can decrease the accumulation of intracellular lipid peroxides and inhibit ferroptosis (27).

**Figure 1:**
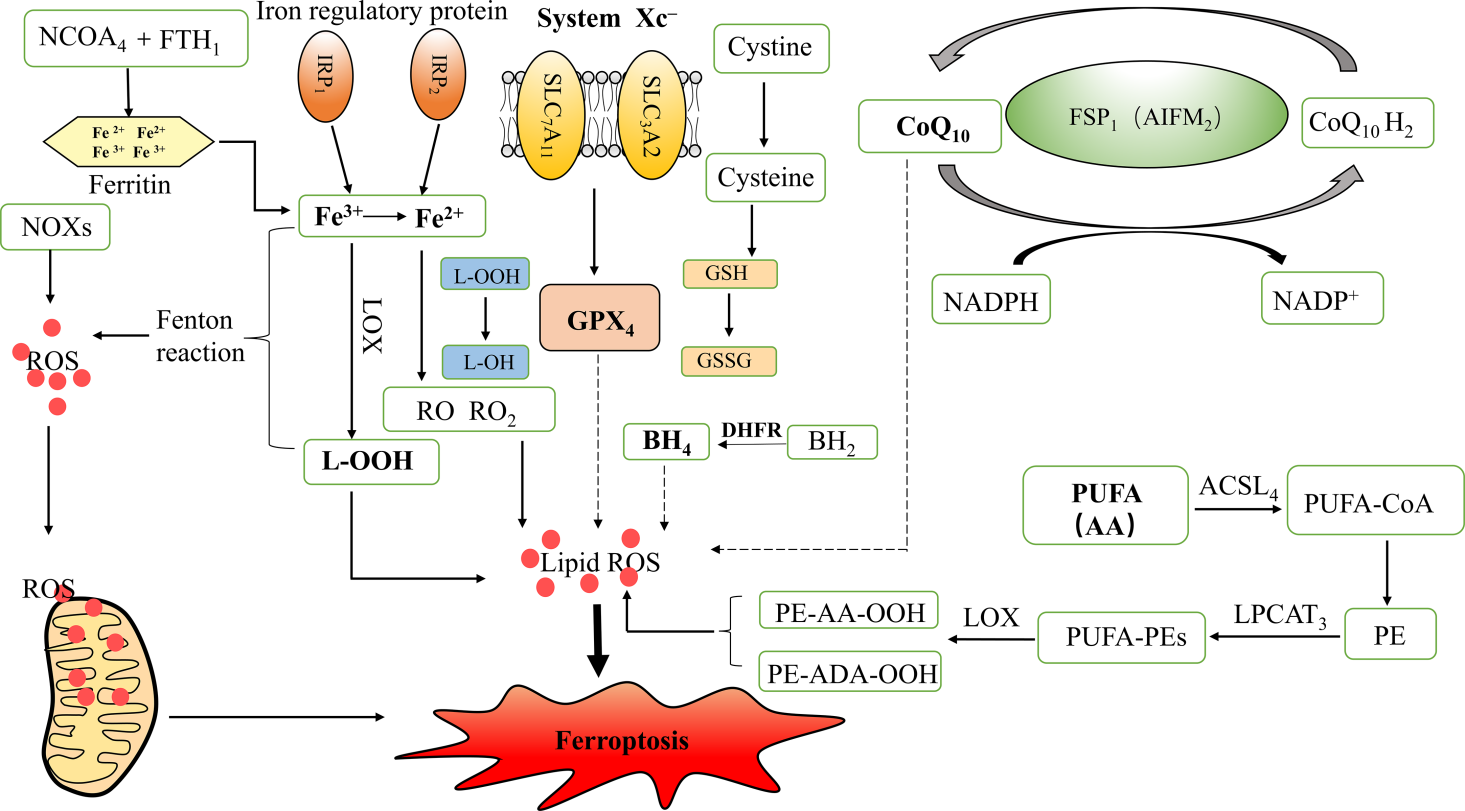
Mechanism of ferroptosis and iron metabolism. ROS, Reactive oxygen species; GPX_4_, Glutathione peroxidase 4; L-OOH, Lipid hydroperoxides; L-OH, Alcohol; RO, Alkoxy; RO_2_, Peroxy; IRP_1_, Iron regulatory protein 1; IRP_2_, Iron regulatory protein 2; LOX, Lipoxygenase; NADPH, Nicotinamide adenine dinucleotide phosphate; NOXs, NADPH Oxidases; GSH, Glutathione; GSSG, Oxidized GSH; SLC_7_A_11_, Solute carrier family 7 members 11; SLC_3_A_2_, Solute carrier family 3 member 2; CoQ_10_, Coenzyme Q_10_/Ubiquinone; CoQ_10_H_2_, Ubiquinol; FSP_1_, Ferroptosis suppressor protein 1; AIFM_2_, Apoptosis inducing factor mitochondrial associated 2; BH_4_, Tetrahydrobiopterin; BH_2_, Dihydrobiopterin; DHFR, Dihydrofolate reductase; PUFA, Polyunsaturated fatty acids; AA, Arachidonic acid; ADA, Adrenic acid; PE, Phosphatidyl ethanolamine; ACSL_4_, Acyl CoA synthase long-chain family member 4; LPCAT_3_, lysophosphatidylcholine acyltransferase 3; PUFA-CoA, Polyunsaturated fatty acid acyl-coenzyme A; PUFA-PEs, PUFA- phosphatidyl ethanolamines; NCOA_4_, Nuclear receptor co-activator 4; FTH_1_, Ferritin heavy chain1.

Autophagy is a fundamental cellular homeostatic program, and excessive autophagy may trigger cell death, also known as autophagy-dependent cell death (18). Recent studies have shown that ferritinophagy is a unique form of selective autophagy mediated by nuclear receptor coactivator 4 (NCOA_4_) (10). Ferritinophagy promotes ferroptosis, which may be attributed to the iron overload caused by increased NCOA_4_ expression (28). NCOA_4_ mediates the degradation of ferritin after combining with ferritin heavy chain 1 (FTH_1_) as a selective autophagic receptor and converts ferritin-bound iron into free iron, thereby inducing ferroptosis (29) (**Figure 1**).

## 3 Typing and diagnosis of diabetic cardiomyopathy and its pathogenesis

### 3.1 Typing and diagnostic criteria of diabetic cardiomyopathy

The histological features of DCM include myocardial collagen deposition, myocardial hypertrophy, and fibrosis (30). It is conventionally believed that the early stages of DCM are characterized by left ventricular (LV) hypertrophy and diastolic dysfunction, whereas the later stages progress to cardiac fibrosis and systolic dysfunction, eventually leading to HF (31). In 1954, Lundbaek first described DCM as a cardiomyopathy that affects two-thirds of elderly patients with diabetes mellitus (32). Two DCM phenotypes, HF with preserved LV ejection fraction (HFpEF) and HF with reduced LV ejection fraction (HFrEF), were identified based on the predominance of cardiomyocyte hypertrophy or apoptosis, the most common of which is the HFpEF phenotype (30).

The minimum diagnostic criteria for DCM include LV diastolic dysfunction and/or reduced LV ejection fraction (LVEF), LV hypertrophy, and interstitial fibrosis and can be divided into three stages: early, advanced, and end. DCM progresses from an initial subclinical phase, characterized by mild structural and functional abnormalities, then to severe diastolic HF with normal ejection fraction (EF), and eventually to HF with systolic dysfunction accompanied by a reduced EF value (33). One of the earliest features of DCM is abnormal LV wall stiffness, mainly secondary to extracellular matrix and myocardial cell remodeling. Cardiac magnetic resonance imaging (MRI) shows weakened ventricular wall motion in all components of myocardial deformation (longitudinal, radial, and circumferential strain). Reduced myocardial strain can also be observed in asymptomatic patients and in T2DM patients without other signs of etiologic heart disease. Therefore, reduced myocardial strain can be considered as a marker of preclinical DCM (30).

### 3.2 Pathophysiology of diabetic cardiomyopathy

Glyco-lipotoxicity, IR, inflammation, oxidative stress, mitochondrial dysfunction, endoplasmic reticulum stress (ERS), and impaired intracellular Ca^2+^ handling may represent the molecular bases for DCM development. In addition, hyperglycemia-related epigenetic modifications may also play an important role in the development of DCM. Among them, histone modifications, especially histone deacetylases, are key regulators of cardiac fibrosis and myocardial hypertrophy (34). The synthesis of microRNA (miRNA) molecules may also be influenced by high glucose levels, and miRNA can affect inflammatory processes and cardiomyocyte survival by regulating oxidative stress response (35).

Oxidative stress can interfere with the balance between antioxidant capacity and ROS production, which is a core mechanism in the development of DCM, and a key regulator of myocardial fibrosis. Myocardial fibrosis is an important feature of cardiac remodeling in DCM (36). Wang et al. reported the induction of the pre- and early stages of DCM using a high-fat diet combined with T2DM rats induced by different doses of streptozotocin (STZ). The results showed increased ROS production in the low- and high-dose STZ treatment groups, with significantly increased cardiac weight index and myocardial collagen deposition in the high-dose group compared to the untreated group. In other words, cardiac structural remodeling, ROS production, and cell death are dysregulated in the pre- and early stages of DCM (37). Excessive ROS in cardiomyocytes leads to mitochondrial DNA damage, lipid peroxidation, post-translational modification of proteins, and eventually cell death and HF (38). ROS production activates redox-sensitive transcription factors and signal transduction pathways, such as protein kinase C (PKC), p38 mitogen-activated protein kinase (p38 MAPK), NH_2_-terminal Jun kinase (JNK), Janus kinase, and signal transducer and activator of transcription (JAK-STAT) pathways, further leading to impaired cardiac structure and function (39). ROS are mainly derived from NOXs, which are oxidative enzymes in the heart, particularly NOX_2_ and NOX_4_. NOX_2_-dependent signaling promotes several deleterious processes in cardiac pathology, including cardiomyocyte hypertrophy, contractile dysfunction, interstitial fibrosis, and cell death. In contrast, NOX_4_ plays important roles in endogenous detoxification reactions, angiogenesis, ERS, substrate utilization, and stress responses (40).

Increased oxidative stress in the heart is also due to cytoplasmic ROS-induced exacerbation of mitochondrial ROS production, and oxidative damage to the mitochondria further causes mitochondrial dysfunction (36). When mitochondrial ROS production exceeds endogenous clearance capacity, it leads to oxidative stress and inflammation. In addition, increased mitochondrial FA uptake and FA β-oxidation in DCM may induce the accumulation of toxic lipid metabolites, resulting in cardiac lipotoxicity and mitochondrial dysfunction (41). It has been recently shown that upregulation of A-kinase anchoring protein 1 (Akap_1_) may have the potential to treat myocardial injury in patients with DCM, where Akap_1_ expression is reduced in a diabetic mouse heart model. Thus, Akap_1_ deficiency may exacerbate DCM deterioration, increase mitochondrial ROS levels, and impair mitochondrial function. In contrast, cardiac-specific overexpression of Akap_1_ restores mitochondrial function and alleviates diabetes-induced cardiac dysfunction and myocardial fibrosis by ectopically regulating NADPH-CoQ in the mitochondria and decreasing mitochondrial ROS (42).

ERS and abnormal Ca^2+^ handling processes are associated with diastolic dysfunction in DCM. In patients with diabetes, persistent hyperglycemia and IR induce ERS and impair Ca^2+^ handling. However, impaired Ca^2+^ handling leads to an increased action potential duration, which consequently causes diastolic dysfunction (41). ERS causes increased myocardial ROS and impaired insulin signaling through activation of the JNK pathway, which is dependent on cellular redox status (43). Cardiomyocyte contractility is affected by impaired insulin signaling and reduced glucose uptake by cardiomyocytes. Additionally, Ca^2+^ is released from cardiomyocytes *via* the ryanodine receptor (RyR), which improves the susceptibility to oxidative stress injury induced by abnormal insulin metabolism and ROS production. Thus, it impairs Ca^2+^ efflux through L-type calcium channels, resulting in a reduction in efflux Ca^2+^ and an increase in intracellular Ca^2+^, which affects the systolic and diastolic functions of cardiac myocytes (44).

Altered cardiac substrate metabolic pathways and impaired energy metabolism are important factors in the pathogenesis of DCM. Under physiological conditions, the heart can employ FA and glucose as energy substrates. In contrast, in diabetic patients, the decrease in glucose uptake due to systemic and cardiac IR promotes a shift of substrates toward increased oxidation of free FA (FFA), resulting in reduced efficiency of cardiac metabolism. The heart loses the ability to utilize glucose, resulting in glucose overload in cardiomyocytes, which promotes the formation of advanced glycation end-products (AGEs) (30, 31). AGEs may play a key role in the development of DCM by stimulating collagen expression and accumulation and promoting collagen cross-linking, which causes increased myocardial fibrosis, reduced myocardial compliance, and impaired myocardial diastolic function (2). In addition, AGEs can also lead to increased intracytoplasmic ROS through activation of the AGE receptor (RAGE), thereby resulting in cardiac diastolic dysfunction (45). Activation of RAGE involves nuclear factor-κB (NF-κB) and its target genes, causing a shift from the α to the β isoform of myosin heavy chain in cardiac myocytes, which reduces myocardial contractility (46).

Alterations in the energy matrix of the heart enable increased FA uptake by cardiomyocytes to exceed the oxidative capacity of mitochondria, leading to excessive lipid storage and production of lipotoxic metabolites that impede cardiomyocyte metabolism and contractility, thereby promoting cardiomyocyte death (47). Excessive accumulation of FA in cardiac tissue and lipotoxicity can also impair insulin signaling, causing reduced cardiac metabolic capacity, increased myocardial oxygen consumption, and abnormal cardiac morphology and structure, ultimately resulting in a significant reduction in cardiac diastolic function (31). In cardiac tissues, the accumulation of lipid metabolites results in the reduced expression of glucose transporter 4 (GLUT_4_) and reduced translocation of GLUT_4_ from the cytoplasm to the cell membrane. As a result, glucose uptake by cardiomyocytes is significantly reduced. On the one hand, it may promote cardiac remodeling; on the other hand, it may inhibit cardioprotective pathways (48). Hyperglycemia and IR activate the PKC signaling pathway, allowing for an increase in myocardial endothelial cell permeability, resulting in endothelial dysfunction. In the pathogenesis of DCM, there is an imbalance in the release of vasoconstrictors [for example, prostanoids, endothelin, and angiotensin-II(Ang-II)] and diastolic agents [for example, nitric oxide (NO), prostacyclin (PGI_2_), bradykinin, and endothelium-derived hyperpolarizing factor (EDHF)] (49). NO, PGI_2_, and EDHF are released from coronary artery endothelial cells, allowing vasodilation. In the early stages of DCM and IR, NO-induced vasodilation is impaired and EDHF-mediated vasodilation is usually maintained or even enhanced to maintain a normal vascular tone. However, in later stages, both NO- and EDHF-induced vasodilation may eventually be impaired, resulting in microvascular dysfunction (41).

**Figure 2:**
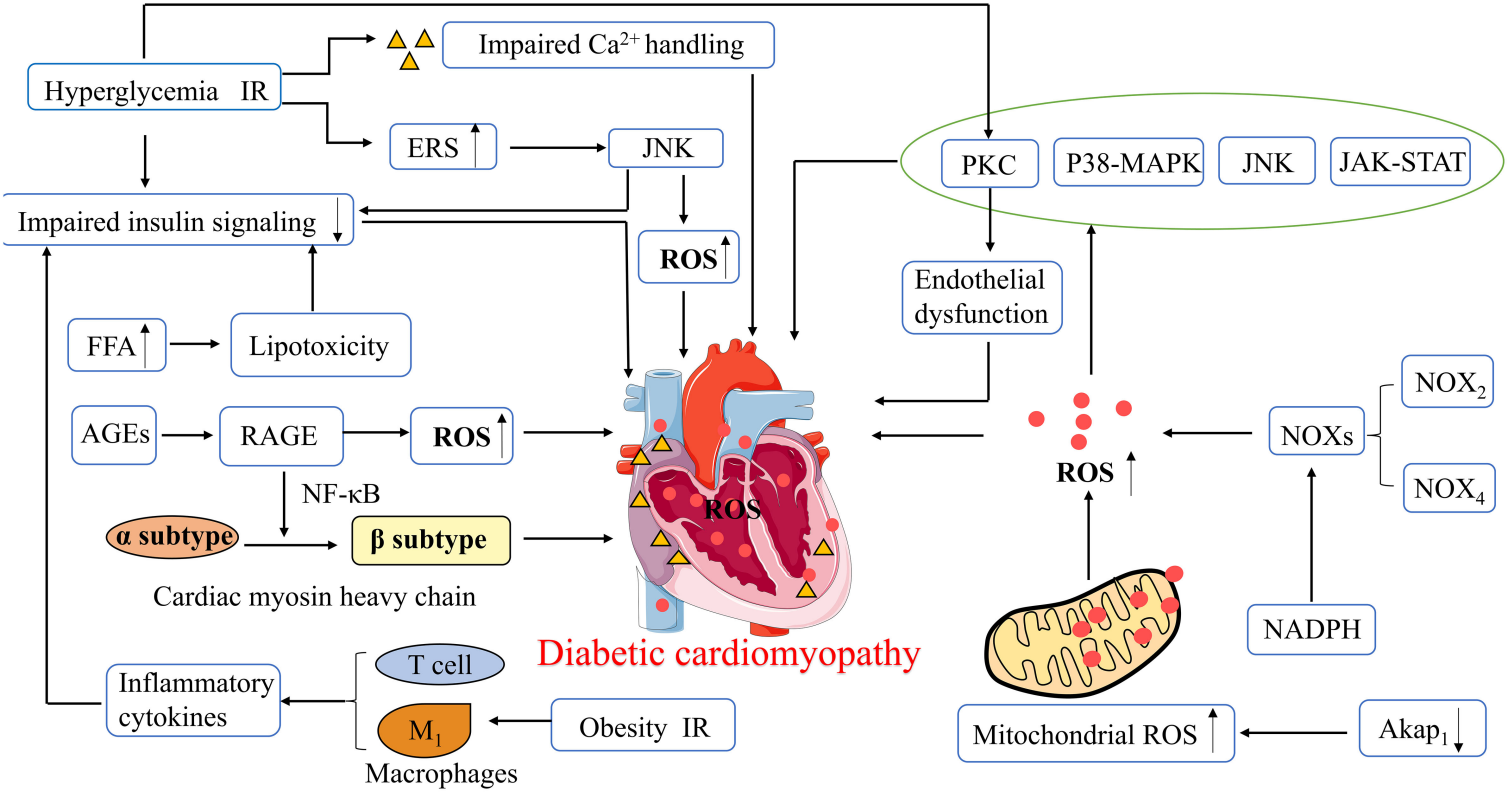
Pathophysiological mechanisms of diabetic cardiomyopathy. ROS, Reactive oxygen species; ERS, Endoplasmic reticulum stress; PKC, protein kinase C; p38 MAPK, p38 Mitogen activated protein kinase; JNK, Jun kinase; JAK -STAT, Janus kinase and signal transducer and activator of transcription; NADPH, Nicotinamide adenine dinucleotide phosphate; NOXs, NADPH Oxidases; Akap_1_, A-kinase anchoring protein 1; FFA, Free fatty acids; AGEs, Advanced glycation end-products; RAGE, AGE receptor; NF-κB, nuclear factor-κB; IR, Insulin resistance.

Diabetes mellitus is an inflammatory disease, and increased ROS levels induce an increase in inflammatory factors. Cardiac inflammation in DCM is mostly caused or exacerbated by increased ROS, and this pro-inflammatory state is largely caused by activation of ROS-triggered inflammatory vesicle nucleotide-binding oligomerization domain-like receptor pyrin domain containing 3 (NLRP_3_) (39). Furthermore, in obesity and IR, M1 macrophages polarize and secrete inflammatory cytokines, causing reduced cardiac insulin signaling and promoting DCM development. Conversely, M2 macrophages secrete IL-10, which inhibits cardiomyocyte hypertrophy and cardiac fibrosis (33). T-lymphocyte inducers contribute to an increase in pro-fibrotic cytokine levels in mouse heart tissue. In addition, T-helper lymphocytes secrete pro-inflammatory cytokines, which can bring about cardiac oxidative stress and coronary artery dysfunction, ultimately leading to cardiac remodeling, fibrosis, and diastolic dysfunction (50). C-reactive protein (CRP) is a cardiovascular pathogenic factor and well-known indicator of inflammation. Mano et al. showed that diabetic mice with CRP overexpression have increased levels of pro-inflammatory cytokines (IL-6 and TNF-α), increased type I collagen, increased expression of brain natriuretic peptide (BNP), and reduced LVEF on echocardiography compared to non-diabetic mice. This means that CRP overexpression may exacerbate LV function, cardiac remodeling, and myocardial fibrosis in DCM patients, possibly through inflammation and oxidative stress (51). Thus, systemic and local adaptive immune responses and inflammation may bring about alterations involving myocardial structure and metabolism, which in turn may cause diastolic dysfunction and eventually HF (**Figure 2**).

## 4 Role of ferroptosis in DCM

It has been shown that cardiomyocytes in diabetic patients are extremely sensitive to cell death, and their cardiomyocyte mortality is 85 times higher than that of non-diabetic patients. Consequently, cell death significantly affects the development of diabetes and its complications. Apoptosis is the main cause of cell death in DCM and promotes DCM cardiomyocyte injury through multiple upstream signaling pathways (52). However, the role of autophagy in DCM pathogenesis of DCM is unclear. Numerous studies have shown that autophagy protects cardiomyocytes in DCM; however, it has also been shown that autophagy can trigger cardiomyocyte injury. In a fructose-induced T2DM diabetic mouse model, upregulated autophagy was associated with elevated cardiac superoxide production and fibrosis, suggesting that autophagy may contribute to DCM (53). In addition, inhibition of various signaling pathways associated with necrosis may reduce myocardial damage in patients with diabetes (52). Ferroptosis is a specific type of cell death characterized by iron dependence and lipid peroxidation, which allows for increased levels of ROS and may promote the occurrence and development of DCM.

Dysregulation of internal environmental homeostasis and iron metabolism is closely related to the development of cardiac diseases, and iron overload is significantly detrimental to the development of ferroptosis and HF in cardiomyocytes (54). Studies have shown that ferroptosis may cause mitochondrial damage in the heart through iron overload, including abnormal mitochondrial structure, altered mitochondrial membrane potential, and increased mitochondrial ROS, which are also considered important features of ferroptosis (55). Iron overload in diabetic patients not only increases the risk of IR and diabetes progression but may also exacerbate cardiovascular complications through the Fenton response (56). DCM development is primarily associated with excessive ROS production and impaired antioxidant capacity. However, the high correlation between ferroptosis and lipid ROS production suggests that the inhibition of ferroptosis may be an important target for DCM prevention and treatment. Abnormal mitochondrial ferroptosis is observed in the hearts of diabetic mice, mainly manifested by decreased mitochondrial membrane potential, downregulated expression of key enzymes involved in antioxidant defence (superoxide dismutase [SOD_2_] and glutathione peroxidase 1 [GPX_1_]) in the mitochondria, and significantly increased mitochondrial ROS levels (57). Another study showed that mice fed a high-iron diet can cause severe myocardial damage and have a typical molecular profile of ferroptosis, including increased lipid peroxidation and reduced GSH levels (58). Thus, iron overload has a detrimental effect on cardiomyocyte function.

In the hyperglycemic state, oxidative stress and impaired antioxidant systems underlie DCM pathogenesis. Ferroptosis, which causes an imbalance between oxidation and antioxidation, typically causes the excessive production of ROS. This is clinically important because cardiomyocytes are highly susceptible to oxidative damage, and lipid peroxidation is involved in ROS-induced cardiac injury. Sampaio et al. reported increased expression of oxidative stress-carbonyl protein markers and myocardial fibrosis (type III collagen) in iron-overloaded T1DM diabetic rats (59). Ghosh et al. showed that GSH was reduced and ROS levels were increased in cardiomyocytes in an STZ diabetic rat model (60).

Glucose-induced persistent cardiomyocyte peroxide accumulation triggers ferroptosis and results in cellular damage at the whole-organism and cellular levels (61). In addition, during DCM onset, the energy metabolism of cardiomyocytes shifts from glycogenolysis to FA oxidation, resulting in increased intracellular lipid accumulation and lipotoxicity (62). However, ferroptosis involves multiple metabolic processes including iron metabolism, lipid metabolism, and in particular lipid peroxide production (63). Therefore, removal of lipid peroxide may attenuate cardiomyocyte injury.

A recent study suggested that the inhibition of ferroptosis by the activation of nuclear factor-erythroid 2-related factor 2 (NRF_2_) may represent a potential therapeutic target in DCM. NRF_2_ regulates multiple antioxidants and plays a key role in maintaining cellular redox reactions (64). NRF_2_ and its target genes have antioxidant, anti-inflammatory, anti-apoptotic, anti-ferroptosis, and anti-fibrotic functions that protect islet β-cells from high-glucose-induced oxidative damage in DCM (65).

Rutin, an NRF_2_ activator and phytochemical, has multiple pharmacological activities, including hypoglycemic and antioxidant activities. In a diabetic mouse model, rutin improved glucolipid metabolism in diabetic mice, attenuated myocardial damage caused by oxidative stress, including ventricular hypertrophy, ventricular remodeling, and ventricular dysfunction, and prevented the progression of myocardial fibrosis, thereby effectively alleviating DCM (66). Additionally, some key regulators of ferroptosis are also the downstream targets of NRF_2_, such as SLC_7_A_11_ and ferritin. SLC_7_A_11_ transports the precursor of GSH, cystine, into the cell matrix, and ferritin plays an important role in iron metabolism by storing excess cellular iron and alleviating the Fenton reaction (67). Almost all genes associated with ferroptosis are transcriptionally regulated by NRF_2_, including GSH-regulated and NADPH-regenerating genes that are essential for GPX_4_ activity, lipid peroxidation, and iron metabolism. The NRF_2_/Kelch-like-epichlorohydrin (ECH)-associated protein 1 (KEAP_1_)/antioxidant response element (ARE) pathway is the main mechanism of myocardial defence against oxidative damage in diabetes and hyperglycemia. It regulates the expression of several genes, most of which are associated with the reduction of oxidative stress and cell death (68). In addition, it has also been shown that the NRF_2_/ferroportin 1 (FPN_1_) signaling pathway is a key mechanism in diabetic myocardial injury, inhibiting ferroptosis by regulating iron metabolic homeostasis, and its activation alleviates diabetic myocardial injury to some extent (64). Furthermore, most patients with diabetes have abnormal lipid metabolism and produce excess saturated FA such as palmitic acid (PA), which plays a role in cardiomyocyte death and the development of DCM. Ferroptosis is associated with PA-induced myocardial injury, and ferroptosis inhibitors significantly reduce cell death in H9c2 and rat cardiomyocytes exposed to PA (10).

BH_4_ inhibits ferroptosis by inhibiting lipid peroxidation. BH_4_ is synthesized by GTP (guanosine triphosphate) cyclohydrolase 1 (GCH_1_) and acts as a cofactor in a variety of biosynthetic pathways, such as the synthesis of aromatic amino acids, neurotransmitters, and NO (69). Overexpression of GCH_1_ protects the heart from DCM and improves cardiac remodeling and dysfunction in a T1DM mouse heart model; therefore, GCH_1_ may serve as a new target for DCM therapy (70).

The increase in oxidative stress generated by iron overload through the Fenton reaction promotes the formation of AGEs, leading to lipid peroxidation, which is an important mechanism in DCM pathogenesis. In an ACE-treated diabetic rat model, ferroptosis inhibitors prevented the diastolic dysfunction of DCM, indicating an important role of ferroptosis in DCM. AGEs induce ferroptosis in engineered cardiac tissues (ECT), as evidenced by increased levels of unstable iron and lipid peroxides and decreased levels of GSH and SLC_7_A_11_ (67).

Endosomal sorting complexes required for transport (ESCRT) play multiple roles in membrane bending or outgrowth as multi-subunit machinery in the material transport process. Various cellular processes, including cell death, are affected by this membrane-remodeling mechanism. In particular, ESCRT-III initiates membrane repair to prevent various types of cell death, including necrosis, apoptosis, and ferroptosis. Activation of ESCRT-III on cell membranes requires Ca^2+^, confirming the role of calcium homeostasis and ERS in the control of ferroptosis (71). Abnormal calcium homeostasis and ERS are also important mechanisms in DCM development. ERS is a cellular response to endoplasmic reticulum dysfunction that can be induced by ROS, and inhibition of ferroptosis alleviates diabetic myocardial ischemia/reperfusion injury, which may provide a new therapeutic target for DCM. ROS are produced by the interaction between Fe^2+^ and NADPH oxidase during ferroptosis. ERS is characterized by the activation of the transcription factor 4 (ATF4)- CCAAT-enhancer-binding protein (C/EBP) homologous protein (CHOP) pathway. It has been shown that, in a diabetic rat model (high glucose and myocardial injury), the diabetic myocardial injury group showed increased levels of ACSL_4_, ATF_4_, and CHOP, severe impairment of cellular arrangement, cell swelling, and most of the myocardial fibers were broken compared to normal control group animals. However, when ferroptosis inhibitors were added, the degree of myocardial injury was significantly reduced in rats, and the levels of ROS, intracellular Fe^2+^ concentration, ACSL_4_, ATF_4_ and CHOP decreased, indicating that ferroptosis inhibitors can improve diabetic myocardial injury by reducing ERS (72).

**Figure 3:**
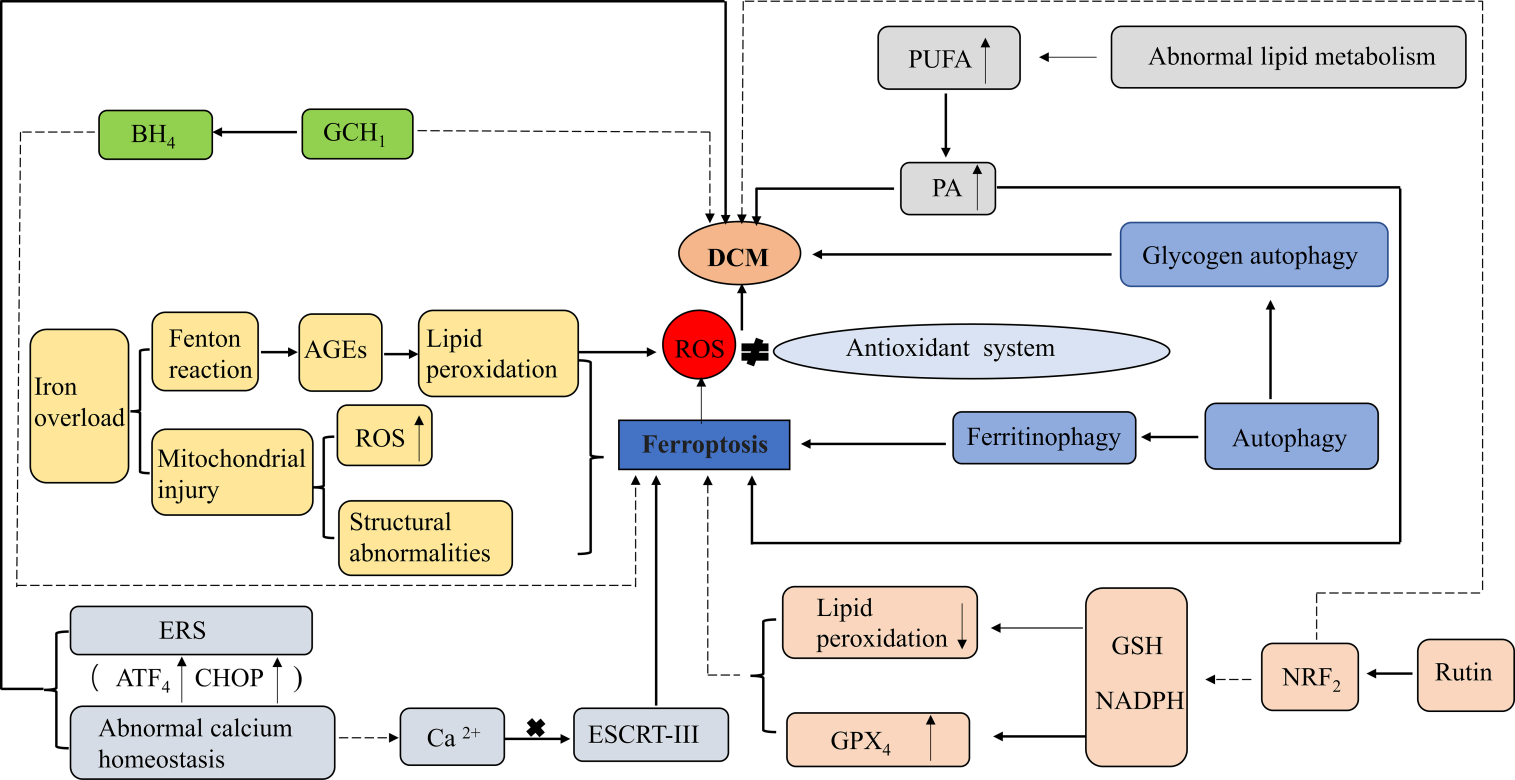
Role of ferroptosis in DCM. ROS, Reactive oxygen species;GPX_4_, Glutathione peroxidase 4; GSH, Glutathione; NADPH, Nicotinamide adenine dinucleotide phosphate; NRF_2_, nuclear factor-erythroid 2-related factor 2; PUFA, Polyunsaturated fatty acids; PA, palmitic acid; AGEs, Advanced glycation end-products; BH_4_, Tetrahydrobiopterin; GCH_1_GTP, (Guanosine triphosphate) cyclohydrolase 1; ERS, Endoplasmic reticulum stress; ESCRT-III, Endosomal sorting complexes required for transport III; ATF_4_, the activation of the transcription factor 4 (ATF4)- CCAAT-enhancer-binding protein (C/EBP) homologous protein (CHOP). Activation: Inhibition.

Autophagy is a fundamental intracellular homeostatic process. Appropriate autophagy may be a pro-survival response; however, excessive autophagy, especially selective autophagy, and impaired lysosomal activity may promote ferroptosis. NCOA_4_-dependent ferritinophagy promotes ferroptosis by releasing free iron from ferritin (73). Many autophagic vesicles have been observed in cardiomyocytes of diabetic patients (74). However, in diabetic mice, 1,25-dihydroxyvitamin-D3 [1-25(OH)_2_VitD_3_] improved myocardial fibrosis and cardiac function in DCM through an autophagy-related vitamin D receptor (VDR)-dependent mechanism and the β-catenin/T-cell factor/lymphoid enhancer factor (TCF_4_)/glycogen synthase kinase-3β (GSK-3β)/mammalian target of rapamycin (mTOR) pathway (75). Glycogen autophagy plays an important role in DCM pathogenesis. In cultured primary cardiomyocytes, glycogen autophagy is regulated by extracellular glucose and insulin and occurs simultaneously with glycogen accumulation (76). In addition, glycogen autophagy is associated with a higher risk of heart disease (including DCM) in female patients, possibly because glycogen autophagy is associated with selective glycogen accumulation in the female myocardium, in which estrogen may upregulate the expression of signaling intermediates that promote glycogen storage (77) (**Figure 3**).

In STZ-induced T1DM mice, the heart showed significant features of DCM, such as myocardial systolic and diastolic dysfunction, impaired Ca^2+^ handling, and myocardial fibrosis (78). The inhibition of autophagy may allow an imbalance in NRF_2_-mediated metabolism as well as redox regulation, which increases iron deposition and lipid peroxidation, promoting cardiomyocyte ferroptosis and the progression of T1DM cardiomyopathy (79). However, in high-fat diet + STZ-induced T2DM mice, the heart is mainly characterized by myocardial steatosis, impaired systolic function, and mitochondrial dysfunction (78). Abnormal glucose and lipid metabolism causes the accumulation of AGEs in the extracellular matrix of the heart, leading to iron overload and lipid peroxidation, which ultimately induce ferroptosis in cardiomyocytes (67). The performance of ferroptosis in different animal models of diabetes is summarised in **Table 1**. Although ferroptosis has been shown to significantly improve cardiac function in animal models and cultured cells, no clinical trials have been performed to date; therefore, population-based studies are needed to determine whether selective blockade of ferroptosis improves the prognosis and/or outcomes of DCM.

**Table 1:** The performance of ferroptosis in different types of animal models of diabetes.

Animal models	Mechanism	Effects on the heart	Biochemical characteristics	References
STZ-induced T1DM mouse model	The inhibition of autophagy may allow imbalance of NRF_2_-mediated metabolism as well as redox regulation, which increase the iron deposition and lipid peroxidation, promoting cardiomyocyte ferroptosis and the progression of T1DM cardiomyopathy	Cardiac fibrosis, hypertrophy, and cardiomyocyte death	Iron deposition, the increased levels in 4-HNE and ACSL_4_, and decreased levels in GPX_4_ and FSP_1_	(79)
High-fat diet + STZ-induced T2DM mouse model	Abnormal glucose and lipid metabolism cause the accumulation of AGEs in the extracellular matrix of the heart, leading to iron overload and lipid peroxidation, which ultimately induce ferroptosis in cardiomyocytes	Myocardial injury, decreased cardiac function, and cardiac remodeling (hypertrophy and fibrosis)	Significantly increased levels in the labile iron, MDA and PTGS_2_, and decreased levels in SLC_7_A_11_ expression and GSH levels	(67)

STZ, Streptozotocin; T1DM, Type 1 diabetes mellitus; T2DM, Type 2 diabetes mellitus; NRF_2_, Nuclear factor-erythroid 2-related factor 2; AGEs, Advanced glycation end products; 4-HNE, 4-hydroxy-2-nonenal; ACSL_4_, Acyl CoA synthase long-chain family member 4; GPX_4_, Glutathione peroxidase 4; FSP_1_, Ferroptosis suppressor protein 1; MDA, Malondialdehyde; PTGS_2_, Prostaglandin-endoperoxide synthase-2; SLC_7_A_11_, Solute carrier family 7 members 11; GSH, Glutathione.

## 5 Potential applications of ferroptosis inducers and inhibitors in DCM

Ferroptosis is promoted by class I (e.g., erastin) and class II (e.g., Ras-selective lethal 3 [RSL-3]) inducers, which indirectly and directly inhibit GPX_4_ activity, respectively (80). Erastin induces ferroptosis by inhibiting system Xc^-^, indirectly decreasing GPX_4_ activity, and directly targeting voltage-dependent anion channels 2 and 3 (VDAC2/3). In contrast, class II RSL-3 promotes ferroptosis primarily by inhibiting the endogenous lipid ROS inhibitor GPX_4_ (81).

Ferroptosis inhibitors act by inhibiting iron accumulation and reducing iron overload (e.g., deferoxamine [DFO]) or by inhibiting lipid peroxidation and reducing lipid ROS production (e.g., liproxstatin-1, vitamin E, and ferrostatin‐1 [Fer-1]) (7).

DFO has been shown to ameliorate cardiomyocyte injury in an *in vitro* DCM model of ECT exposed to AGE, reducing the expression of the lipid peroxidation marker malondialdehyde (MDA) and the ferroptosis marker prostaglandin-endoperoxide synthase-2 (PTGS_2_), improving ECT cardiomyocyte function. Liproxstatin-1 alleviated the decreased diastolic function 3 months after the onset of diabetes, further demonstrating the importance of ferroptosis in the pathogenesis of DCM (67).

Vitamin E is not only a ferroptosis inhibitor but also an endogenous antioxidant defence factor that plays an important role in DCM. Hamblin et al. found that the expression of two myocardial markers of oxidative stress, 8-iso-prostaglandin F2α (8-iso PGF2α) and GSSG, was increased, whereas that of LVEF was decreased in STZ-induced type I diabetic rats. However, after vitamin E supplementation, myocardial oxidative stress decreased and hemodynamic function was enhanced, further demonstrating the role of myocardial oxidative stress in DCM (82).

Fer-1, a potent inhibitor of ferroptosis, acts *via* lipid peroxidation. Treatment with moderate to high doses of Fer-1 reduced ACSL_4_ levels and enhanced GPX_4_ levels, thus reducing mitochondrial ROS production, alleviating mitochondrial dysfunction, and improving LV function in rats with cardiac injury (83). Herceptin (trastuzumab), a human epidermal growth factor receptor 2 (Her-2) gene-related targeted therapeutic agent for the treatment of breast cancer, also exerts toxic effects on the heart. H9c2 rat cardiomyocytes treated with herceptin exhibited decreased GPX_4_ and SLC_7_A_11_ expression with increasing doses of herceptin, inducing H9c2 cardiomyocyte injury, oxidative stress, mitochondrial dysfunction, and ferroptosis. However, the addition of Fer-1 restored GPX_4_ and SLC_7_A_11_ expression levels, which were otherwise inhibited by herceptin, and reversed the herceptin-induced increase in ACSL_4_ expression and increased mitochondrial ROS and iron levels, protecting H9c2 cardiomyocytes from herceptin-induced cardiomyocyte injury and ferroptosis (84).

Heat shock factor 1 (HSF_1_) is a stress-inducible transcription and defence factor against ferroptosis in cardiomyocytes that acts through the transcriptional activation of various heat shock proteins (HSP). A recent study found that PA induced cell death in cardiomyocytes in a dose- and time-dependent manner. Excess unoxidized PA in cardiomyocytes induces oxidative stress, mitochondrial dysfunction, and ceramide accumulation, whereas HSF_1_ significantly inhibits the death of H9c2 and rat cardiomyocytes exposed to PA by regulating the expression of GPX_4_ (10). The potential applications of ferroptosis inducers and inhibitors in DCM are summarised in **Table 2**.

**Table 2:** Potential applicationsof ferroptosis inducers and inhibitors in DCM.

		Animal/Cell	Mechanisms and effects on the heart	References
Inducers	Erastin	Male wild-type mice	Erastin caused cardiomyocyte death by inhibiting system Xc^-^, indirectly decreasing GPX_4_ activity and directly targeting VDAC2/3	(81)
	RSL-3	Male wild-type mice	RSL-3 caused cardiomyocyte death by inhibiting the endogenous lipid ROS inhibitor GPX_4_	(81)
Inhibitors	DFO	FVB mice and wild-type mice	DFO ameliorated cardiomyocyte injury by reducing iron overload and lipid peroxidation	(67)
	Liproxstatin-1	FVB mice and wild-type mice	Liproxstatin-1 alleviated the decrease in diastolic function at 3 months after the onset of diabetes by inhibiting lipid peroxidation	(67)
	Vitamin E	Sprague-Dawley rats	Vitamin E improved cardiac systolic and diastolic function by reducing oxidative stress	(82)
	Fer‐1	MaleWistar rats/H9c2 rat cardiomyocytes	Fer-1 reduced ACSL_4_ levels and enhanced GPX_4_ levels by inhibiting lipid peroxidation, which further improved mitochondrial oxidative stress and reduces myocardial injury.	(83, 84)
	HSF_1_	H9c2cardiomyocytes	HSF_1_ reduced myocardial injury from oxidative stress by regulating GPX_4_ expression	(10)

RSL-3, Ras-selective lethal 3; VDAC2/3, Voltage-dependent anion channels 2 and 3; DFO, Deferoxamine; Fer-1, Ferrostatin‐1; ACSL_4_, Acyl CoA synthase long-chain family member 4; GPX_4_, Glutathione peroxidase 4; ROS, Reactive oxygen species; HSF_1_, Heat shock factor 1.

## 6 Conclusions

An increasing number of studies have confirmed the relationship between ferroptosis and metabolic diseases, one of the more serious complications of diabetes mellitus, mainly through mechanisms such as glucolipotoxicity, inflammatory responses, and oxidative stress, resulting in increased myocardial tissue fibrosis and impaired systolic and diastolic functions. Among these, ROS overproduction may be considered an important bridge between the two. Large amounts of ROS promote oxidative stress and damage cardiac myocytes, which in turn leads to myocardial systolic and diastolic dysfunction. Thus, ferroptosis may be a new therapeutic target for diabetic cardiomyopathy. However, the role of ferroptosis in DCM is still poorly understood, and in-depth clinical studies are lacking. In addition, clinically reliable and sensitive markers for ferroptosis in early DCM are needed. Finally, objective DCM diagnostic criteria are still lacking, making it difficult to determine whether myocardial injury, hemodynamic changes, and decreased cardiac function caused by ferroptosis should be considered DCM or possibly other cardiovascular diseases, such as coronary atherosclerosis or ischemic cardiomyopathy.

## Author contributions

All authors contributed to the study conception and design. The first draft of the manuscript was written by YZ, and all authors commented on previous versions of the manuscript. All authors contributed to the article and approved the submitted version.
